# Positive Effect of α-Asaronol on the Incidence of Post-Stroke Epilepsy for Rat with Cerebral Ischemia-Reperfusion Injury

**DOI:** 10.3390/molecules27061984

**Published:** 2022-03-18

**Authors:** Lan Jiang, Xiangnan Hu

**Affiliations:** 1 College of Environment and Resources, Chongqing Technology and Business University, Chongqing 400067, China; jianglan@ctbu.edu.cn; 2 Department of Medicinal Chemistry, School of Pharmacy, Chongqing Medical University, Chongqing 400016, China

**Keywords:** post-stroke epilepsy, ischemic stroke, α-asaronol, n-butylphthalide

## Abstract

In the present study, we confirmed that α-asaronol, which is a product of the active metabolites of alpha Asarone, did not affect n-butylphthalide efficacy when n-butylphthalide and α-asaronol were co-administered to rats with cerebral ischemia-reperfusion injury. Our research revealed that the co-administration of α-asaronol and n-butylphthalide could further improve neurological function, reduce brain infarct volume, increase the number of Nissl bodies, and decrease the ratios of apoptotic cells and the expression of the caspase-3 protein for cerebral ischemia-reperfusion injury model compared to n-butylphthalide alone. Additionally, α-asaronol could significantly decrease the incidence of post-stroke epilepsy versus n-butylphthalide. This study provides valuable data for the follow-up prodrug research of α-asaronol and n-butylphthalide.

## 1. Introduction

Stroke, a nervous system disease which is caused by acute cerebral blood circulation disorder, is the second leading global cause of death and a leading cause of long-term disability [1]. It poses a serious threat to human health, with a high incidence, and results in high mortality and disability rates [2]. According to its pathological mechanisms, stroke can be clinically categorized into ischemic and hemorrhagic. Ischemic strokes, accounting for approximately 87% of all strokes, are primarily caused by thrombosis and arterial obstruction [3].

Patients with ischemic strokes are susceptible to a variety of complications, of which epilepsy can further lead to the aggravation of hypoxia/anoxia in brain tissue, ultimately adversely affecting patients’ recovery of motor function [4]. This complication is a common clinical problem and is found in 8.6% of patients with ischemic stroke [5]. In general, seizures following stroke can be divided into early- and late-onset. The former refers to the development of seizures within 2 weeks of suffering stroke, while the latter refers to onset after 2 weeks [6]. It has been reported that early-onset seizure was a risk factor for the development of late-onset seizures following stroke [7]. In addition, status epilepticus in the acute phase of ischemic stroke usually causes the severe dysfunction of the cerebral nervous system, which further increases mortality in patients with ischemic stroke [8,9]. The prevention of seizure after ischemic stroke is highly beneficial to patients; however, the American Heart Association Stroke Council and the European Stroke Organization do not recommend the prophylactic use of anti-epileptic drugs for early- or late-onset seizures after ischemic stroke. This is primarily because of the lack of robust evidence in previous studies [10,11]. The clinical treatment of seizure prevention after stroke by the prophylactic administration of anti-epileptic drugs has led to increased complications and mortality in patients being observed in some studies [12,13]. Additionally, anti-epileptic drugs have many side effects such as drowsiness, impaired memory, and blurred vision [14]. Hence, there is an urgent need to address this problem.

In recent decades, Chinese herbal medicines, which have thousands of years of history of use in therapy, have become increasingly popular in clinical use due to their good safety profiles and few toxic side effects [15,16]. Alpha Asaronem is one of the active components of Acorus calamus, a traditional Chinese medicine which exhibits distinct anticonvulsant and anti-epileptic effects [17,18]. α-asaronol ((E)-3(2,4,5-trimethoxyphenyl)-prop-2-en-1-ol), which is the product of active metabolites of alpha Asarone [19], possesses an even better anticonvulsant activity than alpha Asarone [20]. Racemic 3-n-butylphthalide (NBP), which is a natural compound extracted from the seeds of Chinese celery, is approved by the State Food and Drug Administration of China for the treatment of patients with acute ischemic stroke [21,22]. Given this, we expect that combining α-asaronol (ASOL) and NBP could lead to better outcomes in patients with ischemic stroke by reducing seizures while limiting adverse outcomes.

## 2. Results and Discussion

### 2.1. The Effect of ASOL on mNSS

**Figure 1:**
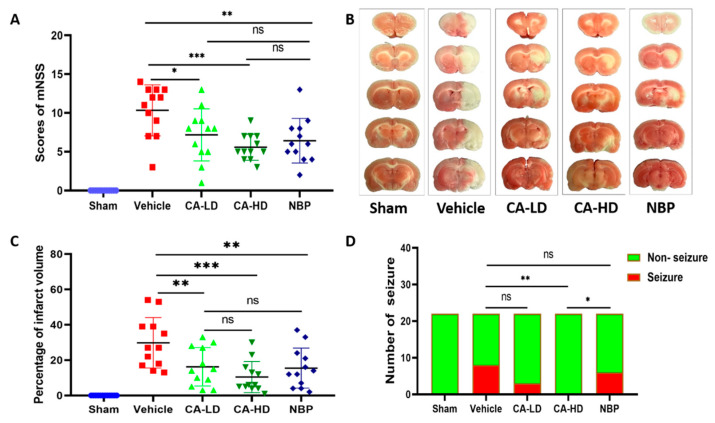
ASOL treatment in the presence of NBP enhanced neurofunctional recovery, reduced infarct volume, and decreased the incidence of post-stroke epilepsy. (**A**) Quantitative analysis results obtained for the neurological score for rats with cerebral ischemia-reperfusion injury (*n* = 12). (**B**) Representative images of coronal brain sections for rats with cerebral ischemia-reperfusion injury with TTC staining. (**C**) The quantitative analysis results of infarct volume for rats with cerebral ischemia-reperfusion injury (*n* = 12). (**D**) Quantitative analysis results of the incidence of post-stroke epilepsy for rats with cerebral ischemia-reperfusion injury (*n* = 22). CA-LD, coadministration of 10 mg/kg NBP and 15 mg/kg ASOL. CA-HD, coadministration of 10 mg/kg NBP and 30 mg/kg ASOL. ns means not significant, * *p* < 0.05, ** *p* < 0.01, *** *p* < 0.001.

In the present study, in order to explore the effect of ASOL in the presence of NBP on cerebral ischemia-reperfusion injury, we investigated a model of cerebral ischemia-reperfusion injury. The vehicle group had the highest score of mNSS among the five groups, which revealed that the modeling was successful (Figure 1A). Compared to the vehicle group, CA-LD (coadministration of 10 mg/kg NBP and 15 mg/kg ASOL), CA-HD (coadministration of 10 mg/kg NBP and 30 mg/kg ASOL), and NBP significantly decreased the score for mNSS (CA-LD *p* < 0.05, CA-HD *p* < 0.001, NBP *p* < 0.01). The difference in the scores of mNSS of CA-LD and CA-HD versus NBP was not statistically significant. Further, the score obtained for CA-HD performed slightly better than that for NBP. These results show that ASOL did not affect NBP efficacy when NBP and ASOL were co-administered. On the other hand, high-dose α-asaronol (CA-HD) was beneficial to the recovery of the neurological function of rats with cerebral ischemia-reperfusion injury.

### 2.2. The Effect of ASOL on Infarct Volume

The un-infarcted area was red and the infarcted area was white in the representative photographs of TTC staining for rat with cerebral ischemia-reperfusion injury (Figure 1B). The results obtained for infarct volume are shown in Figure 1C, which demonstrates that the vehicle group had the highest percentage of infarct volume. It can be seen that CA-LD, CA-HD, and NBP significantly reduced the percentage of infarct volume when compared with the vehicle group (CA-LD *p* < 0.01, CA-HD *p* < 0.001, NBP *p* < 0.01). Furthermore, compared with NBP, there was no statistical significance for CA-LD and CA-HD. Additionally, the infarct volume of CA-HD performed slightly betterthan that for NBP. This further confirmed the suggestion made above that a high dose of α-asaronol (CA-HD) was beneficial for the recovery of rats with cerebral ischemia-reperfusion injury.

### 2.3. The Effect of ASOL on the Incidence of Post-Stroke Epilepsy

Post-stroke epilepsy is a very common complication in stroke survivors [23]; as such, we aimed to test whether ASOL treatment was effective in preventing post-stroke epilepsy for rats with cerebral ischemia-reperfusion injury. As demonstrated in Figure 1D, the highest incidence of post-stroke epilepsy was observed in the vehicle group. CA-LH, CA-HD, and NBP reduced the incidence of post-stroke epilepsy, whereas only CA-HD showed a statistically significant difference compared to the vehicle group (*p* < 0.01). Furthermore, compared with NBP, CA-HD showed a very significant reduction in the incidence of post-stroke epilepsy (*p* < 0.05). It was suggested that ASOL treatment may be an effective means to prevent the occurrence and development of post-stroke epilepsy in the presence of NBP for rats with cerebral ischemia-reperfusion injury.

Post-stroke epilepsy, which accounts for almost 50% of newly diagnosed epilepsy in patients with an age greater than 60 years, could lead to the aggravation of the disease state, seriously affect the prognosis, and even endanger life [24]. However, most antiepileptic drugs that are used to prevent and treat epilepsy have obvious toxic side effects [25]. Interestingly, ASOL possesses a very low toxicity and minimal side effects. Furthermore, the acute toxicity of ASOL is far less than that of α-asarone, which is its parent compound [20]. In a previous study, NBP was shown to exert multi-target pharmacological functions against ischemic stroke, including reducing oxidative stress, inhibiting inflammatory responses, alleviating neuronal apoptosis, and increasing regional blood flow [21]. However, it does not have any antiepileptic effects. Thus, ASOL, which exhibits distinct anticonvulsant and anti-epilepsy effects, is a promising therapeutic drug for the prevention and treatment of post-stroke epilepsy in the future.

### 2.4. The Effect of ASOL on the Results of Nissl Staining

**Figure 2:**
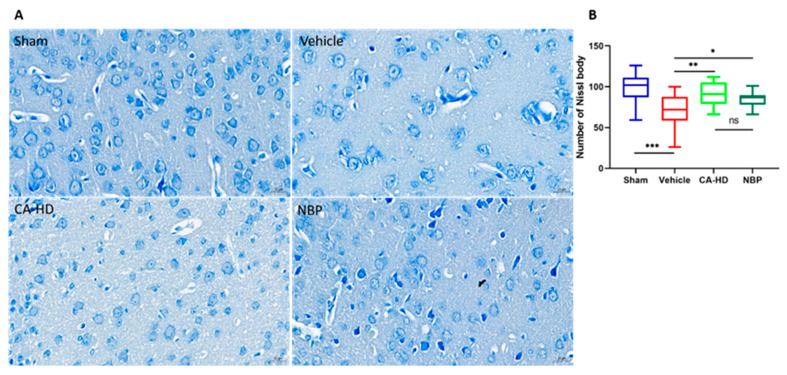
The effects of ASOL treatment in the presence of NBP on the peri-infarct cortical neurons of rats with cerebral ischemia-reperfusion injury. (**A**) Representative images of histological examination by Nissl staining showing the survival of neurons in the peri-infarct cortical region. (**B**) Graphical representations of the data through box-and-whisker plots, where the whiskers indicate the minimum and maximum (*n* = 6). CA-LD, coadministration of 10 mg/kg NBP and 15 mg/kg ASOL. CA-HD, coadministration of 10 mg/kg NBP and 30 mg/kg ASOL. ns means not significant, * *p* < 0.05, ** *p* < 0.01, *** *p* < 0.001.

Nissl bodies are a marker of mature neurons that reflect the functional state of neurons and can be used to detect neuronal apoptosis [26]. In this light, we evaluated the effects of ASOL treatment in the presence of NBP on the peri-infarct cortical neurons of rats with cerebral ischemia-reperfusion injury using Nissl staining. The data (Figure 2) showed that the vehicle group had the lowest number of Nissl bodies in the peri-infarct cortical regions, which was also significantly reduced compared to the sham group (*p* < 0.001). Both CA-HD and NBP showed remarkable increases in the number of Nissl bodies compared with the vehicle group (CA-HD *p* < 0.01, NBP *p* < 0.05). Furthermore, the number of Nissl bodies in CA-HD was shown to be slightly higher than that in NBP. This is consistent with the above findings.

### 2.5. The Effect of ASOL on the Results of TUNEL Staining

**Figure 3:**
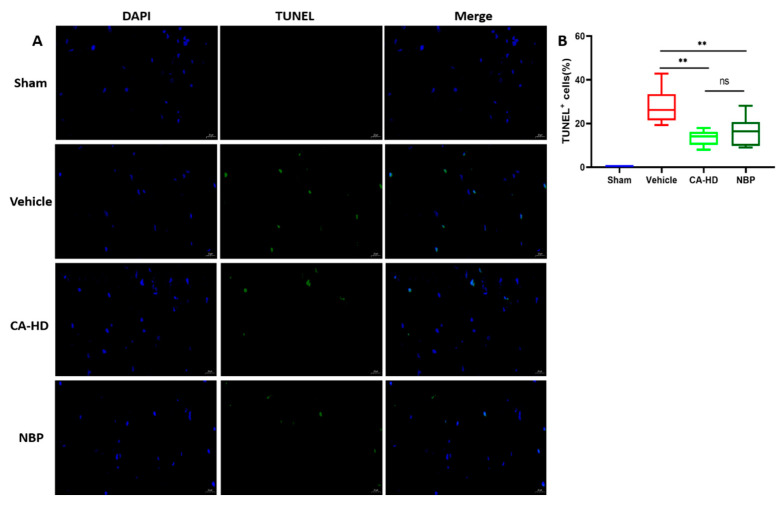
The effects of ASOL treatment in the presence of NBP on the peri-infarct cortical neuron of rats with cerebral ischemia-reperfusion injury. (**A**) Representative images of histological examination by TUNEL staining showing the death of neurons in the peri-infarct cortical region (400-fold visual field). (**B**) Graphical representations of the data through box-and-whisker plots, where the whiskers indicate the minimum and maximum (*n* = 6). CA-LD, coadministration of 10 mg/kg NBP and 15 mg/kg ASOL. CA-HD, coadministration of 10 mg/kg NBP and 30 mg/kg ASOL. ns means not significant, ** *p* < 0.01.

TUNEL staining is a method that is used to detect the DNA fragmentation generated during apoptosis [27]. TUNEL-positive cells exhibit green fluorescence, while TUNEL-negative nuclei show a blue color under DAPI. The data (Figure 3) showed that there were no apoptotic cells in the sham group, and that the ratio of apoptotic cells in the vehicle group was the highest. Treatment with CA-HD or NBP was shown to reduce the ratio of apoptotic cells significantly (<0.01) compared to in the vehicle group. No significant differences were found between the CA-HD and NBP groups. Furthermore, CA-HD exhibited lower mean ratios than the NBP’s for TUNEL-positive cells. These findings indicated that the coadministration of NBP and ASOL further attenuated neuronal damage in the peri-infarct cortex area.

### 2.6. Immunohistochemical Analysis of Caspase-3 Expression

**Figure 4:**
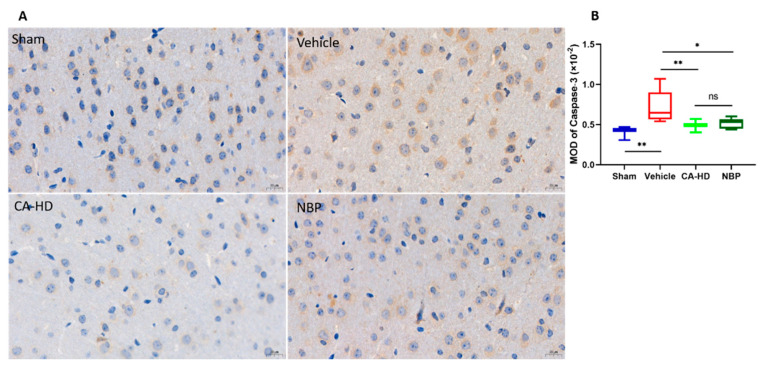
The effects of ASOL treatment in the presence of NBP on the peri-infarct cortical neurons of rats with cerebral ischemia-reperfusion injury. (**A**) Representative images of immunohistochemistry showing the distribution of caspase-3 protein in the peri-infarct cortical region. (**B**) Graphical representations of the data through box-and-whisker plots, where the whiskers indicate the minimum and maximum (*n* = 6). CA-LD, coadministration of 10 mg/kg NBP and 15 mg/kg ASOL. CA-HD, coadministration of 10 mg/kg NBP and 30 mg/kg ASOL. ns means not significant, * *p* < 0.05, ** *p* < 0.01.

The activation and expression of caspase-3, a cysteine protease, preceded neuronal damage, which ultimately leads to cell apoptosis [28,29,30]. Therefore, we evaluated ASOL’s effect on the peri-infarct cortical neurons of rats with cerebral ischemia-reperfusion injury by the immunohistochemical detection of the caspase-3 protein. The more strongly the caspase-3 protein is expressed, the more severe the neuronal damage in the peri-infarct cortical region is. From Figure 4, it can be seen that the caspase-3 expression in the vehicle group was significantly increased when compared to that in the sham group (*p* < 0.01). After the administration of CA-HD and NBP, the expression of the caspase-3 protein significantly decreased (CA-HD *p*< 0.01, NBP *p* < 0.05) compared to that in the vehicle group. There was no significant difference between the CA-HD and NBP groups, but the CA-HD group showed a lower mean optical density than the NBP’s for the expression of caspase-3. These results further confirm that α-asaronol was beneficial to the recovery of rats with cerebral ischemia-reperfusion injury.

## 3. Conclusions

In this study, we firstly confirmed that α-asaronol did not affect the efficacy of NBP when NBP and α-asaronol were co-administered to rats with cerebral ischemia-reperfusion injury. Subsequently, we determined that the co-administration of α-asaronol and NBP could further improve neurological function, reduce brain infarct volume, increase the number of Nissl bodies, and decrease the ratios of apoptotic cells and the expression of the caspase-3 protein compared to NBP alone. More importantly, α-asaronol could significantly decrease the incidence of post-stroke epilepsy versus NBP. These positive results substantiate the notion that α-asaronol treatment is an effective means to prevent the occurrence and development of post-stroke epilepsy. A prodrug could be made via an esterification reaction from hydroxyl groups of α-asaronol and carboxylic groups which the ester hydrolysis of NBP afforded.The results of this study provide a theoretical basis for follow-up prodrug research on α-asaronol and NBP.

## 4. Materials and Methods

### 4.1. Drug

α-asaronol (purity: >99%) was kindly provided by Prof. Hu from Chongqing Medical University. Racemic 3-n-butylphthalide was purchased from Macklin. ASOL and NBP were dissolved in 5–10% Solutol HS-15 and 90–95% saline.

### 4.2. Animals

Male Sprague Dawley rats (200~220 g) were obtained from the Animal Laboratory Center of Chongqing Medical University (Chongqing, China). All animals were housed under a 12 h light–dark cycle ad libitum in a temperature-controlled (25 ± 1 °C) facility and maintained on standard food and water. All animal experimental protocols were in accordance with the guidelines of the National Institutes of Health and approved by the Experimental Ethics Committee of Chongqing Medical University (license number: SYXK (YU) 2010-001).

### 4.3. Middle Cerebral Artery Occlusion (MCAO) and Treatments

All animals were fasted for 10–12 h before the study but had free access to water. All animal experiments were performed in a blinded setup. The MCAO model was established according to the literature with minor modifications [31]. Briefly, animals were anesthetized via intraperitoneal injection with 10% chloral hydrate (350 mg/kg). The right common carotid artery (CCA), right external carotid artery (ECA), and right internal carotid artery (ICA) were exposed and isolated. Then, a 4-0 monofilament nylon suture (Cinontech Co., Ltd., Beijing, China) with silicon coating was inserted into the ICA via the ECA stump and gently advanced to occlude the middle cerebral artery after the CCA and ECA were simultaneously ligated. Zea-longa scores were used to evaluate the neurological function of the SD rats after they had regained full consciousness (post-anesthesia) to verify the success of modeling [32]. Following 2 h of MCAO, the monofilament was gently removed to allow reperfusion. The surgical neck wound was closed immediately with sutures. Next, SD rats in the different groups were intravenously injected with the appropriate drugs or a volume-matched vehicle. Following injection, the animals were placed in the cage for normal feeding. The sham group underwent the same procedures, except for the occlusion of the middle cerebral artery.

### 4.4. Evaluation of the Neurological Deficits

Sixty male adult SD rats (200~230 g) were randomly distributed into five groups (*n* = 12): sham, vehicle, coadministration of NBP (10 mg/kg) and ASOL (15 mg/kg) (CA-LD), coadministration of NBP (10 mg/kg) and ASOL (30 mg/kg) (CA-HD), and administration of NBP (10 mg/kg) only. After 24 h of cerebral ischemia-reperfusion, the neurological deficit scores were assessed blindly according to the modified neurological severity score (mNSS) system [33]. The mNSS test included assessments of four different functions (motor, sensory, reflex, and balance), with the highest possible score being 18. In this scoring system, higher scores reflected more severe injury.

### 4.5. Determination of Infarct Volume

The brain tissue of each group was quickly isolated, frozen for 30 min at −20 °C, and cut into 2 mm-thick coronal sections after a neurological function evaluation. The sections were then stained with 2% 2,3,5-triphenyltetrazolium chloride (TTC; Macklin) in phosphate-buffered saline (PBS) for 30 min in the dark and fixed with 4% paraformaldehyde (PFA) for 24 h. These slices stained with TTC were photographed and the infarction areas were measured using the Image J 1.52p analysis software (National Institutes of Health, Washington, DC, USA). The calculation of the percentage of infarct volume was performed with the sum infarct area of the ipsilateral hemisphere/the sum area of the hemisphere.

### 4.6. Evaluation of Post-Stroke Epilepsy

SD rats (200~230 g) were randomly divided into five groups (*n* = 22): sham, vehicle, coadministration of NBP (10 mg/kg) and ASOL (15 mg/kg) (CA-LD), coadministration of NBP (10 mg/kg) and ASOL (30 mg/kg) (CA-HD), and administration of NBP (10 mg/kg) only. Rats were closely observed for the occurrence of tonic-clonic seizures and status epilepticus in the 24 h after cerebral ischemia-reperfusion and administration.

### 4.7. Brain Tissue Preparation

After conducting post-stroke epilepsy tests on the sham, CA-HD, and NBP groups, six rats were randomly selected from each group. The selected rats were deeply anesthetised by injecting them with 10% chloral hydrate and then perfused with saline, followed by 20 mL of 4% PFA. The brain was removed and then fixed with 4% PFA for 72 h. The fixed brain tissues were dehydrated sequentially in 70%, 80%, and 90% alcohol for 90 min each. Finally, the tissues were paraffin-embedded, and serial coronal sections were sectioned at 5 μm thicknesses for later use [34].

### 4.8. Nissl Staining

Nissl staining was carried out according to the standard practices of pathological examination to measure the number of Nissl bodies in the peri-infarct cortex area. In brief, 5 µm sections were transferred and immersed in 3-aminopropyl-3-ethylylsilane (APES) to closely pack the brain tissues to the slides. After the coronal sections were dewaxed and rehydrated, these sections were stained with a toluidine blue solution, washed with distilled water, soaked in 70% alcohol, and differentiated in 95% alcohol. Sections were then subjected to dehydration with anhydrous ethanol, cleaned with xylene until transparent, and sealed with neutral gum. Nissl staining images were acquired from the peri-infarct cortex area with a Panoramic 250 digital slice scanner (Danjier, Jinan, China) and analyzed via the Image J 1.52p analysis software (National Institutes of Health, Washington, DC, USA). The mean density of neurons was calculated from up to three non-overlapping and representative images obtained from the brain sections of rats from different groups.

### 4.9. TUNEL Staining

TUNEL staining was performed according to the manufacturer’s instructions using the cell death detection kit (Roche, Basel, Switzerland). In brief, after being deparaffinized and rehydrated, the sections were repaired with the citrate repair solution (pH 6.0) and washed with phosphate-buffered saline (PBS). TUNEL staining solution was added and the sections were incubated in dark for 60 min at room temperature. After rinsing, the sections were then stained with DAPI (Leagene Biotech, Beijing, China). Finally, the sections were washed with PBS and sealed with a glycerin gelatin mixture. TUNEL staining images were acquired from the peri-infarct cortex area with a Panoramic 250 digital slice scanner (Danjier, Jinan, China) and analyzed via the Image J analysis software (National Institutes of Health). The TUNEL-positive cells and the total number of nuclei were counted from up to three non-overlapping and representative images obtained from the brain sections of rats from different groups, and the ratios of apoptotic cells were then calculated in each group.

### 4.10. Caspase-3 Activity Determination

After dewaxing sections, the sections were treated with 3% H_2_O_2_ in methanol for 10 min, then washed with PBS for 5 min at RT; this process was repeated three times. Subsequently, the sections were dipped into citrate solution (pH 6.0), heated to boiling, and then cooled at room temperature. After 10 min, the heating and cooling cycle was repeated once. After being cooled to room temperature, sections were washed with PBS twice for 6 min each at room temperature. The sections were blocked with normal goat serum (ZSGB-BIO, Beijing, China) at RT for 25 min and incubated with primary antibody against caspase-3 (rabbit polyclonal antibody, abcam, UK) at 4 °C for 12 h. Then, sections were incubated with the secondary antibody of biotin-labeled goat anti-rabbit (ZSGB-BIO, Beijing, China) at 37 °C for 40 min and washed with PBS three times for 6 min each time. The slides were developed with the DAB chromogen kit (ZSGB-BIO, Beijing, China), their color development was checked under a microscope, and then they were washed with distilled water. Finally, the sections were slightly counterstained with hematoxylin, dehydrated, transparentized, and sealed with neutral gum. Sections were scanned using a Panoramic 250 Digital Slide Scanner (Danjier, China) and analyzed using Image-Pro Plus 6.0 (Media Cybernetics, Rockville, MD, USA). Integrated optical density (IOD) and the positive area of all images in the peri-infarct cortical neurons were measured from up to three non-overlapping and representative images obtained from the brain sections of rats from the different groups. The calculation of the mean optical density (MOD) of each image was performed by dividing the IOD by the positive area.

### 4.11. Statistical Analysis

All data were analyzed using the IBM SPSS Statistics 26.0 software (IBM, New York, MY, USA), and the graphical representations were performed via GraphPad Prism 8.0 software (GraphPad Software, San Diego, CA, USA). If the data followed a normal distribution and the groups had equal variances, multiple comparisons among groups were analyzed via a one-way analysis of variance (ANOVA), followed by LSD post hoc analysis. If the data followed a normal distribution and the groups had unequal variances, multiple comparisons among groups were evaluated by a one-way ANOVA with Dunnett’s T3 test. The incidence of post-stroke epilepsy was analyzed using the chi-square test for comparisons among groups. Values of *p* < 0.05 were considered statistically significant.

## Data Availability

The data presented in this study are available on request from the corresponding author.

## Sample Availability

Samples of the compounds are not available from the authors.
